# 3D Imaging Segmentation and 3D Rendering Process for a Precise Puncture Strategy During PCNL – a Pilot Study

**DOI:** 10.3389/fsurg.2022.891596

**Published:** 2022-05-03

**Authors:** Otaš Durutović, Aleksandar Filipović, Katarina Milićević, Bhaskar Somani, Esteban Emiliani, Andreas Skolarikos, Milica M. Janković

**Affiliations:** ^1^ Faculty of Medicine, University of Belgrade, Belgrade, Serbia; ^2^ Clinic of Urology, University Clinical Centre of Serbia, Belgrade, Serbia; ^3^ Center for Radiology and Magnetic Resonance Imaging, University Clinical Centre of Serbia, Belgrade, Serbia; ^4^ Laboratory for Biomedical Instrumentation and Technologies, Department of Signals and Systems, University of Belgrade, School of Electrical Engineering, Belgrade, Serbia; ^5^ Faculty of Medicine, University Hospital Southampton, Southampton, United Kingdom; ^6^ Department of Urology, Fundacion Puigvert, Autonomous University of Barcelona, Barcelona, Spain; ^7^ National and Kapodistrian University of Athens, 2nd Department of Urology, Sismanoglio Hospital, Athens, Greece

**Keywords:** 3D reconstruction, 3D rendering, PCNL, puncture guidance, kidney stone

## Abstract

Percutaneous nephrolithotomy (PCNL) is frequently used as the first-line treatment of large and complex stones. The key point for successful complex stone removal with minimal risk of complications is to establish the most appropriate access route. Understanding the three-dimensional (3D) relationship of kidney stones and renal collecting systems is crucial for planning and creating an optimal access route. By using a 3D volume segmentation tool a more accurate approach to the renal collecting system and stone treatment could be planned. The objective of this study was assessing the impact of 3D software in getting the desired access.

## Introduction

During renal stone treatment planning, non-contrast computerized tomography (NCCT) is a highly recommended examination, used for assessing the stone size, number, and location. Non-excretory phases do not provide precise insight into detailed stone distribution inside the renal collecting system. With the use of an additional excretory phase scan, the urologist can study in-depth the relationship between the stone and the occupied pelvicalyceal system.

European Association of Urology (EAU) Urolithiasis Guidelines recommend percutaneous nephrolithotomy (PCNL) as the first-line treatment of large stones, more than 2 cm (1). Urologists usually define a renal stone as being complex based on the complicated and difficult pelvicalyceal anatomy, branching of the stone into the pelvicalyceal system, or the existence of multiple stones in various calyces. PCNL treatment of complex renal stones frequently require several access tracts and clear out the stone completely (2–4). However, the overall complication rate, as well as the transfusion rate, is higher when using multiple tracts (5–7). The key point for successful complex stone removal with minimal risk of complications is to establish the most appropriate access route (8). That means that the urologist should select and puncture that calyx(s) which will lead to complete stone extraction, while they will not increase perioperative complication rate.

Understanding the three-dimensional (3D) relationship of kidney stones and renal collecting systems is crucial for planning and creating an optimal access route. In recent years the use of advanced imaging and 3D reconstruction of CT data in the treatment of kidney stones is increasing (8). Advanced imaging systems have been shown to provide the 3D perception of target organs, surrounding anatomic structures, depth perception, and spatial orientation during endoscopic and minimally invasive surgery (9, 10).

There is a continuous search for new and improved methods that could help and refine the course of the PCNL procedure by achieving a more accurate approach to the renal collecting system and stone treatment while decreasing the risk of complications (11).

For the purpose of this study an elective semi-automated instrument was created for a more exact and quantitative evaluation of the renal collecting system and the stone by using a 3D volume segmentation tool. This could help for preoperative comprehensive PCNL access route planning but is also viable for real-time use in the operating room. The aim of this study was to assess the impact of 3D software in getting the desired access.

## Methods

In this pilot case study, we enrolled 5 patients, which were selected with kidney stones, planned for PCNL procedure. CT scans were performed using a 64 multi-detector row CT scanner (General Electric) with 0.625 mm axial plane reconstruction. The scan was performed before and after contrast administration and both phases (plain and excretory) were used for the segmentation process and fusion of the 3D images into a single semi-transparent model demonstrating the stone distribution in the collecting system. While NCCT was used for stone segmentation, the excretory phase was used for collecting system segmentation. The scan delay time for excretory phase after i.v. contrast administration was 10–15 min, depending on the kidney excretion and degree of obstruction if present. The data set was routinely analyzed in Advantage Workstation Software V 4.6. The contrast medium used was Ultravist 370 mg J/mL, approximately 70–100 mL depending on the patient’s weight.

After NCCT acquisition DICOM images were used for segmentation and 3D reconstruction of the renal stones using the *3D Gastro CT Ex tool*. The same software was used for segmentation and 3D reconstruction of pelvicalyceal system images obtained at the excretory phase scan. The reconstructed 3D model represented the interrelation between the pelvicalyceal system and stone with the greater anatomical details. Visualization is achieved by adjusting the scanned object transparency. Segmentations were performed to obtain stone volume (SV) and pelvicalyceal system volume (PSV). After volume rendering of the collecting system and renal stone, the stone volume (SV) was analyzed and compared with the pelvicalyceal system (PS) volume. The volumetric ratio of the stone and collecting system (SV/PS) was also analyzed.

### Software Environment

*3D Gastro CT Ex tool* is an open-source tool developed on Python 3. This tool is the extended version of the *3D Gastro CT* tool that was previously introduced (12). The previous software version supports segmentation and 3D rendering of abdominal CT scans for individual phases (native and vein phase). The new software version, whose advantages in the planning of PCNL treatment are illustrated through this paper, offers the option for 3D hybrid visualization of native and delayed phase, which will be described in detail. The following libraries and toolkits were used in the tool development process: matplotlib (13), ndimage (14), SimpleITK (15–17), and VTK (Visualization Toolkit) (18). PyQt5 binding for Qt v5 was used for designing an intuitive and user-friendly graphical interface (19). The tool offers options for reading and viewing different image formats that are supported by SimpleITK reader (e.g., *Dicom*, *MetaImage*, etc.). Export options for saving rendering results in.stl and .jpg formats are available. The source code of *3D Gastro CT Ex* is available for download from the Github repository: https://github.com/milicevickatarina/3D-Gastro-CT-Extended.

### Image Preprocessing

Image preprocessing of axial CT slices (512 × 512) included band shifting on a histogram, image noise re-movement, and co-registration process. First, the band [−548, 800] was shifted to [0,255] using a linear intensity transfer function. Second, image noise removal was performed by median filtering. Finally, Mattes mutual information was used as a criterion for the co-registration of native and delayed phase using the *SimpleITK* class *Image Registration Method* (sitkLinear was used as interpolator and gradient descent was used for the optimization process) (20).

### Segmentation and 3D Rendering

**Figure 1:**
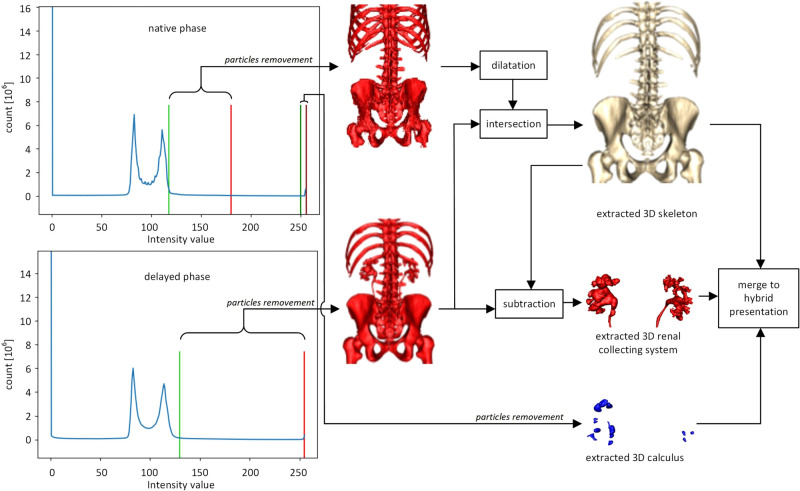
The flow chart of the segmentation and 3D rendering process.

The overall segmentation process on native and delayed phases is presented in **Figure 1**. First, the segmentation procedure implies the calculation of histograms for the whole CT volume of both phases. Skeleton segmentation includes the following steps:

1. Volume binarization from the native phase based on the histogram thresholding method (default threshold values: lower = 117 and upper = 180, light green line and light red line on native phase histogram in **Figure 1** respectively)
2. Particle removal from the volume extracted in step 1 based on opening and closing of the volume (cube structural element with the dimension 3)
3. Dilatation of the volume extracted in step 2
4. Volume binarization from the delayed phase based on the histogram thresholding method (default threshold values: lower = 130 and upper = 255, light green line and light red line on delayed phase histogram in **Figure 1** respectively)
5. Particle removal from the volume extracted in step 3 based on opening and closing of the volume (cube structural element with the dimension 3)
6. The intersection of volumes obtained in step 3 and step 5.

A renal collecting system is extracted by the subtraction of volumes archived in steps 6 and 5.

Calculus segmentation includes the following steps:

1. Volume binarization from the native phase based on the histogram thresholding method (default threshold values: lower = 250 and upper = 255, dark green line and dark red line in **Figure 1** respectively)
2. Particle removal from the volume extracted in the previous step is based on the opening and closing of the volume (cube structural element with the dimension 2).

3D rendering was performed by Marching Cubes algorithm (21, 22). VTK renderer was used for the implementation of rotation and zoom options (**Figure 1**). The processing time of DICOM files required to obtain a semi-automatic 3D image is from 45 to 90 min on a computer with the following features: processor Intel (R) Core (TM) i7-8565U CPU@1.80 GHz, 8GB RAM, graphics card NVIDIA GeForce MX250

At the operating room, the patient was positioned on the table in a prone position with all the coordinates and measurements taken out from both conventional CT evaluation and 3D model translated on the skin of the lumbar area to enable the planned access route. Puncture was performed under ultrasound and pulsed fluoroscopy control after retrograde contrast injection through the ureteral catheter. Tract was dilated up to 30 Fr Amplatz sheath (standard PCNL) with the use of 26 Fr nephroscope.

## Results

**Figure 2:**
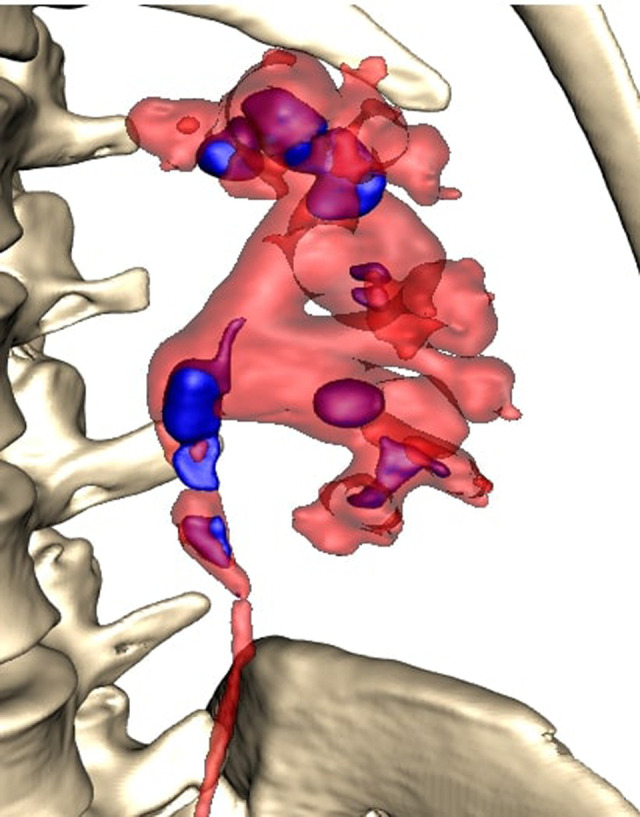
Anterior view.

**Figure 3:**
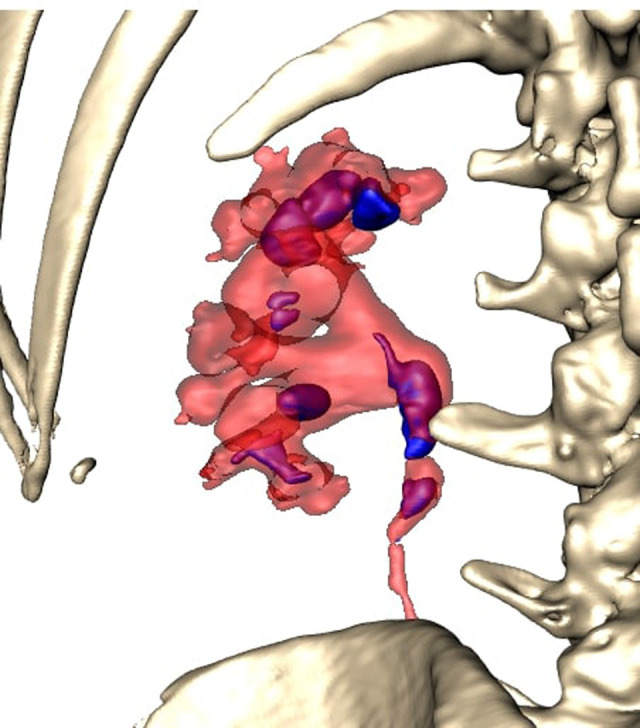
Posterior view.

**Figure 4:**
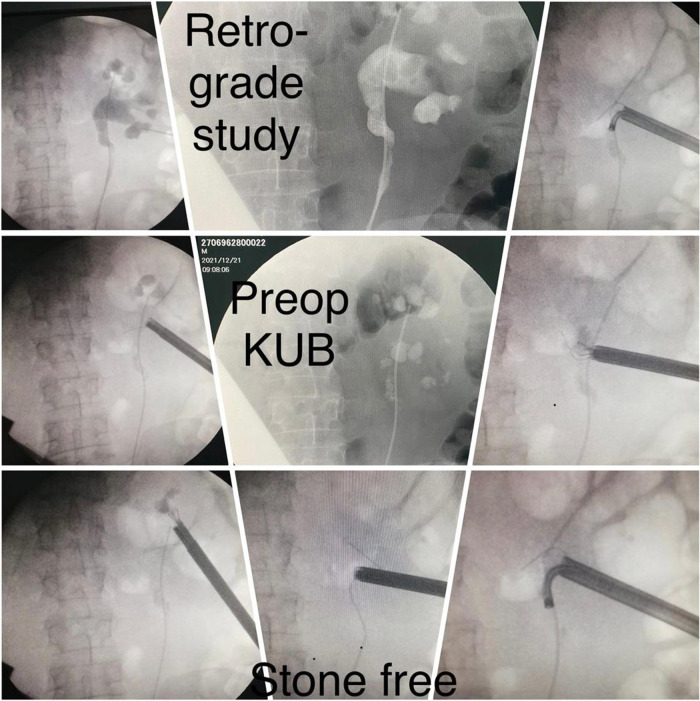
Images from surgery.

All patients were completely investigated by conventional protocols proposed by guidelines. After confirming the indication for surgery, an additional puncture strategy was made after 3D reconstruction using the 3D Gastro CT Ex tool. Images from conventional CT scanners were compared with new images and an optimal axis for percutaneous puncture was made upon case characteristics. Due to the 3D reconstructed semi-transparent images, it was possible to analyze volumetric datasets in both anterior and posterior view (**Figures 2 and 3**). Patients from the study group had complex renal stones (Guy stone score 3 and 4) (23). All cases were treated by using a single puncture (**Figure 4**). The use of flexible nephroscope was indicated by case characteristics and surgery flow. Stone-free status was achieved in all cases, confirmed by follow up imaging. No perioperative complication occurred. In our series there was no unsuccessful outcome of PCNL procedures performed regardless the use of the 3D software for puncture planning.

## Discussion

The pelvicalyceal system with a complex stone represents a complex 3D structure. Thus, for accurate access route planning, the semi-transparent 3D model representing the correlation between the collecting system and stone seems to be very useful.

Rendering of the CT images with the ability to adjust the transparency of different tissue layers allowed the operator to see the stone through the renal collecting system, locate the stone and navigate the puncture needle. This semi-transparent 3D model could provide additional information during endourological procedures and therefore raise the possibility of achieving one-stage clearance of complex renal stones. The greatest advantage of using a 3D software is in creating a clear preoperative plan and selecting a target access route. Once having a clear preoperative puncture plan further steps of puncture guidance and control can be performed very precisely, usually with less fluoroscopy use. In this way the total fluoroscopy time can be reduced. As the total fluoroscopy time is a surrogate for radiation exposure, we assume that preoperative planning is a good way to also achieve the goal of reducing the use of fluoroscopy to a minimum in accordance with ALARA principles.

It has been proven that the surgeon is more comfortable with the initial puncture due to the 3D preoperative planning (24, 25). PCNL is routinely performed under 2D visualization with ultrasound and/or fluoroscopy guidance. In our technique the use of flexible nephroscope is not mandatory but flexible nephroscope should always be available during PCNL in aim to clear all stones from the pelvicalyceal system but also upper ureter.

By using a 3D model of CT images in planning the route and controlling the needle path, we would be able to use all the advantages of multidetector CT (MDCT) without raising the risk of radiation dose as it would be the case with CT fluoroscopy or Cone-beam CT guidance. 89% of success rate for percutaneous kidney access has been reported when using Cone-beam CT for guidance, but it comes with a higher radiation dose than conventional fluoroscopy guidance (26).

An accurate preoperatively defined puncture trajectory may also contribute to decreasing total fluoroscopy time to the lowest possible level. Once the operator has a clear preoperative puncture plan during fluoroscopy and ultrasound-guided PCNL procedures, fluoroscopy can be used only for access tract formation and wire position confirmation. Shortening of fluoroscopy time during PCNL is an important technical requirement to ensure safety and efficiency in PCNL while also reducing the radiation exposure of the patient and surgical team (11).

Our puncture technique involves ultrasound guidance with the assistance of pulsed fluoroscopy. Compared with our initial cases which were performed by ultrasound and continuous fluoroscopy we have already achieved significant reduction in fluoroscopy time from 155.4 s (median value) to 76.8 s (27).

If we compare the total fluoroscopy time values of PCNL procedures performed without the use of 3D software already reported in our larger series (27) with the total fluoroscopy time values of the PCNL procedures performed in this study (after creating the semi-transparent 3D model of pyelocaliceal system and stone) a significant reduction in the total fluoroscopy time is noted when 3D software was used (76.8 s vs 27.7 s).

Bearing in mind that in this study the results obtained on a small sample of five patients are presented, we cannot speak from the relevance of the statistically obtained data. This reduction can also be a result of an increased experience and skills of surgeon (OD), as his volume increased during the years.

To this study, CT images were segmented by focusing on renal stone, collecting system, and bone structures serving as a landmark for translation of measurements on the patient’s skin. Contrast is not absolutely mandatory for MDCT examination, especially in cases with damaged renal function. As the majority of patients have normal renal function the use of contrast offers a huge benefit in term of detail determination and distinction between stone and pelvicalyceal system.

Measurements of puncture angles and distances from the referent points were collected from a 3D model and translated in the operating room with accuracy. Vascular structures’ segmentation was not taken into consideration because the initial puncture of the renal collecting system was performed by an experienced urologist with real-time ultrasound guidance. The vessel segmentation process could take additional time before the procedure starting point without any substantial decrease in the rate of vascular injuries.

From the point of PCNL safety, avoidance of unnecessary puncture(s) is crucial. A greater rate of complications has been reported during calyceal stones treatment in comparison with the treatment of pelvic stones (28). When planning treatment with rigid instruments, the access route should be as straight as possible from the skin through the calyx in the axial line of the calyx through the infundibulum of the certain calyx and into the renal pelvis. Any additional intraoperative angulation of the access sheath would increase the risk of renal injury and bleeding. Every percutaneous kidney puncture is associated with a risk of bleeding. With the need for tract formation and dilatation, this risk is increased. The transfusion rate is higher in cases where multiple tracts were used during PCNL. Therefore, a decrease in access tracts is one of the goals of endourologists. The use of flexible nephroscope can decrease the need for additional puncture. In our opinion, even with flexible nephroscope the importance of optimal access axis remains of huge importance. Optimal access offers two biggest benefits: avoidance of torqueing and in that way less possibility of bleeding and better visibility during the procedure, especially in complex cases with stone distribution in different calices. Another benefit of detailed preoperative planning and selection of an optimal access route is in possibility to easily approach to all other calices that contain stones (such as in parallel calices, that sometimes can not easily be approached, even with flexible nephroscope from the punctured calyx) or might be a possible place of stone migration (usually upper calyx).

Stone burden and volume distribution in the renal collecting system is proven to be a significant determinant of the stone-free rate (SFR) (24), which was defined as no residual fragments larger than 3 mm in diameter (29, 30). In our initial experience and case series, the use of this tool helped significantly in preoperative planning and surgical strategy, allowing access to secondary calices from the main axis tract more easily.

There were no additional costs for this kind of evaluation, the software is free for use and its utilize was simple and intuitive. Collaboration with radiologists and software engineers was necessary, especially as the program offers the opportunity of rotation of the image and creating a picture in all positions used for PCNL, whether prone or supine or Valdivia modified used for retrograde intrarenal surgery (RIRS).

The limitation of the study was a shift and change in the position of the stone that can happen with the positioning of the patient. This was especially the case with smaller stones in the dilated collecting system. After 3D model calculation and taking measurements in a few cases the stone had changed its position most likely due to patient positioning. The CT scan was routinely performed while the patient was in the supine position and the PCNL procedure was performed in a prone position.

## Conclusion

Having a clear and precise preoperative puncture plan is the key point to ensure efficiency in PCNL. Optimal access according to stone distribution and renal collecting system anatomy may contribute to both PCNL procedure efficacy and safety. This semi-automated segmentation tool for 3D visualization of the collecting system and stone interrelation is proven to be very useful for preoperative PCNL access planning. Currently, this might be a time-consuming process, but in the future, it should be a completely automated process with widespread use and adoption for all complex stone procedures.

## Data Availability

The datasets presented in this study can be found in online repositories. The names of the repository/repositories and accession number(s) can be found in the article.
